# Among the Colleges

**Published:** 1902-03

**Authors:** 


### AMONG THE COLLEGES.

The Grand Rapids Veterinary College has established a stock farm in connection with the college. Here they purpose raising and keeping on hand a great variety of animals and birds for use in demonstrations and instructions among the students. The *Michigan Veterinary Review* will again resume publication in connection with the college, but under the management of a company.

Dr. William A. McLean has succeeded Dr. William C. Wooton as dean, while Dr. L. L. Conkey, D.V.S., M.F., is general manager of the college. It is confidently believed by those identified with the institution that all these changes will inure to the general good of the college.

The Western Veterinary College has plans out for a commodious college building.

The operating-table at the Kansas City Veterinary College attracted much attention during the meeting of the State Association. Its special points of value—compactness, portability, lightness, strength, cheapness, and ease of manipulation—are generally admitted.

Dr. E. M. Nighbert, of Mt. Sterling, Illinois, and Dr. Frank Linscott, of Holton, Kansas, are matriculates in the third-year class of the Kansas City Veterinary College.

The January meeting of the Students' Society of the University of Pennsylvania Veterinary Department was addressed by Dr. W. Horace Hoskins on "Future Outlook of the Profession." Almost the entire student body were present and evinced a keen interest in the bright future of the veterinary profession as portrayed by the speaker.

